# A Comparative Analysis of Raw and Bran-Fried *Acori tatarinowii Rhizoma* Based on the Intelligent Sensory Evaluation System

**DOI:** 10.3390/metabo15050338

**Published:** 2025-05-20

**Authors:** Yingna Le, Zhongjian Yang, Ruiping Wang, Shaolong Ma, Yang Cui, Kun Shi, Li Xin, Jinlian Zhang, Lingyun Zhong

**Affiliations:** 1 School of Pharmacy, Jiangxi University of Chinese Medicine, No. 1688, Meiling Avenue, Xinjian District, Nanchang 330004, China; leyingna@ncmc.edu.cn (Y.L.); shikun@jxutcm.edu.cn (K.S.); xinli@jxutcm.edu.cn (L.X.); 2 School of Pharmacy, Nanchang Medical College, Nanchang 330052, China; 202202006089@ncmc.edu.cn (Z.Y.); wangruiping@ncmc.edu.cn (R.W.); cuiyang@ncmc.edu.cn (Y.C.); 3 Research and Development Department, Jiangzhong Pharmaceutical Co., Ltd., Nanchang 330004, China; shaolong272005@126.com

**Keywords:** *Acori tatarinowii Rhizoma*, bran-fried, Heracles NEO ultra-fast gas-phase electronic nose, electronic eye, electronic tongue, quality control

## Abstract

**Objectives:** The study aimed to investigate the differences in odor, color, and taste characteristics between raw and bran-fried *Acori tatarinowii Rhizoma* (RATR and BATR) using advanced sensory evaluation technologies. The objective was to establish a reliable differential analysis method for distinguishing RATR and BATR slices to support quality control in herbal processing. **Methods:** The Heracles NEO ultra-fast gas-phase electronic nose was employed to analyze odor profiles, while electronic eye and electronic tongue technologies were used to assess color and taste differences, respectively. Odor fingerprint analysis identified key volatile components, and colorimetric and taste measurements were conducted to compare RATR and BATR samples. **Results:** Fifteen characteristic odor components were identified, with methanol, 2-propanol, and 2-cyclopentenone potentially serving as discriminant markers differentiating RATR and BATR. PCA demonstrated exceptional separation efficacy, with a cumulative contribution rate of 99.937% for the primary components. **Conclusions:** The integration of Heracles NEO electronic nose, electronic eye, and electronic tongue technologies effectively distinguished RATR from BATR. This approach provides a novel strategy for online quality monitoring in herbal slice production and offers a robust analytical framework for the identification and quality assessment of processed herbal medicines.

## 1. Introduction

*Acori tatarinowii Rhizoma* (ATR) is the dried rhizome of *Acori tatarinowii Schott*, a plant belonging to the Araceae family. This herb has bitter, pungent, and sour flavors and is associated with the spleen and stomach meridians. It is primarily produced in Jiangxi, Sichuan, Hunan, Hubei, and Guizhou provinces [1]. ATR contains various compounds, such as phenylpropanoids, terpenoids, lignans, flavonoids, alkaloids, amides, and organic acids [2]. The processing methods for ATR mainly include bran-fried ATR, fresh ATR, and ginger-processed ATR. The clinical applications of ATR change after bran-frying, highlighting the importance of accurately distinguishing between different processed forms of traditional Chinese medicinal slices for scientific and rational clinical use [3].

Odor, color, and taste are among the significant characteristics in the traditional empirical identification of Chinese medicine. After being stir-fried with bran, the odor of ATR shifts from ‘aromatic’ to ‘having a roasted aroma’, and its color changes from ‘off-white or slightly red’ to ‘yellowish-brown to dark brown on the surface’. The changes in taste before and after processing are not explicitly described in the local standards [4]. And these descriptions are somewhat subjective and vague. The electronic nose is a device that simulates the human olfactory system. It detects odors through a specific array of sensors and uses a pattern recognition system to analyze complex scents [5]. The Heracles NEO ultra-fast gas-phase electronic nose is an analytical instrument that combines dual-column fast gas chromatography with chemometric pattern recognition for volatile organic compound (VOC) analysis. This system utilizes gas chromatographic separation principles with dual columns (weak and medium polarity) and dual FID detectors to achieve enhanced compound separation [6]. Characteristic chromatographic fingerprints are generated and analyzed through principal component analysis and discriminant factor analysis, enabling multivariate discrimination of odorant substances [7]. The technology demonstrates capabilities in rapid VOC detection and analysis, with published studies reporting its application in food quality assessment, pharmaceutical analysis, and fragrance characterization [8,9]. Comparative studies with conventional GC-MS and GC-IMS techniques have shown this approach can provide both compound identification and sensory-relevant information through the integration of chromatographic data with odor threshold detection [10,11,12].

Electronic sensory evaluation systems primarily consist of an E-nose, E-eye, and E-tongue, which are capable of assessing the odor, color, and taste of samples. Advanced sensory analysis technology, based on human sensory bionics, integrates sensor arrays with data processing units and pattern recognition systems to detect samples [13]. This technology offers advantages such as high precision and excellent reproducibility, providing a scientific and data-driven complement to traditional sensory evaluation methods. It is increasingly being applied in the production and development of food and pharmaceutical products [14,15,16,17]. Zhang et al. tracked the odor changes in ginger-processed Magnolia officinalis and found decreased magnolol/honokiol content but increased relative levels of (−)-α-pinene and β-pinene [18]. Zhang et al. employed an electronic eye (EE) combined with chemometrics to analyze characteristic features and quality control of honey-processed Glycyrrhiza uralensis prepared with different refined honeys. The powdered samples were photographed on a visual analyzer to obtain chromaticity values. Results demonstrated positive correlations between the liquiritin/isoliquiritigenin content and total color value, with significant differences observed between honey-fried products in both morphological characteristics and chemical composition, providing references for quality evaluation standards [19]. Li et al. utilized an electronic tongue (E-tongue) to analyze taste variations among raw, stir-fried, charred, and carbonized Crataegi Fructus samples [20].

This study integrates Heracles NEO ultra-fast gas-phase electronic nose, electronic eye, and electronic tongue technologies to analyze the differences in odor components, colorimetric variations, and taste profiles of RATR and BATR. The aim is to distinguish between raw and bran-fried ATR slices based on odor, color, and taste, providing a reference for quality control of ATR medicinal materials.

## 2. Materials and Methods

### 2.1. Materials

ATR was purchased from Kangmei (Bozhou) Huatuo International Traditional Chinese Medicine City Commercial Co., Ltd. (Bozhou, China). All raw ATR materials were stored in vacuum-sealed aluminum foil bags (with silica gel desiccant) at 4 °C for a maximum of 72 h before processing. Detailed sample batch information was provided in Table 1. The n-alkane mixture reference standards (nC6 to nC16) were obtained from Restek Corporation (Bellefonte, PA, USA), batch number A0200647.

### 2.2. Preparation of Raw and Bran-Fried ATR

Ten batches of ATR medicinal materials were prepared, with detailed information provided in Table 1. These included raw products (R1–R5) and bran-fried ATR slices (B1–B5), which were processed under the ATR section and the general rules for bran-frying, according to the methods outlined in *Chinese Pharmacopoeia* [21]. Precisely weighed raw ATR slices (3.00 kg, 3 mm thickness) were processed in an electromagnetic stir-frying machine (DCCY-900, Hangzhou Jinzhu Co., Hangzhou, China) upon observation of wheat bran smoke (0.45 kg). The frying was maintained at 180 °C (ST20 Infrared Thermometer, Raytek, Santa Cruz, CA, USA) for 4.0 min with continuous stirring (30 rpm). The processed slices were immediately sieved (2 mm mesh), transferred to enamel trays, and cooled under controlled ambient conditions (25 °C, 60% RH).

### 2.3. Preparation of Test Samples

The raw and bran-fried ATR samples were prepared for analysis using the Heracles NEO ultra-fast gas-phase electronic nose system. The samples were first ground to a fine powder using a laboratory grinder (Model BO-400Y multifunctional pulverizer, Yongkang Bo’ou Hardware Factory, Yongkang, China) and passed through a no. 5 sieve (180 μm mesh) to ensure particle size uniformity. The homogenized powders were then stored in airtight containers at 4 °C for a maximum of 72 h until analysis. For electronic nose analysis, aliquots of 0.25 g of each powdered sample were accurately weighed into 20 mL headspace vials. The vials were immediately sealed with aluminum crimp caps equipped with silicone septa to prevent volatile loss. All samples were prepared in triplicate to ensure analytical reproducibility. Prior to analysis, the sealed vials were equilibrated at 45 °C for 5 min in the automated sampler to allow for volatile compound accumulation in the headspace.

### 2.4. Establishment of the Odor Detection Method for Raw and Bran-Fried Acori tatarinowii Rhizoma

To achieve optimal separation and analytical performance, the detection conditions were optimized by investigating key factors, including incubation temperature, incubation time, sample amount, and injection volume. Raw *Acori tatarinowii Rhizoma* powder was used as the test sample for single-factor experiments.

#### 2.4.1. Optimization of Incubation Temperature

Precisely weighed 0.25 g of sample R5 was analyzed under fixed conditions (incubation time: 5 min, injection volume: 500 μL) at different incubation temperatures (35, 40, 45, 50, and 55 °C). Each condition was tested in triplicate. Chromatographic analysis revealed that the chromatograms obtained at 45 °C exhibited the most abundant peak information with good stability and peak shape. Therefore, 45 °C was selected as the optimal incubation temperature.

#### 2.4.2. Optimization of Incubation Time

Precisely weighed 0.25 g of sample B5 was tested at 45 °C with an injection volume of 500 μL while varying the incubation time (1, 3, 5, 7, and 9 min). Each condition was analyzed in triplicate. The results indicated that peak areas increased with prolonged incubation time. After 5 min, the chromatographic peaks reached saturation and stabilized. Thus, 5 min was chosen as the optimal incubation time. Notably, when the incubation time increased from 3 to 7 min, the peak area at 70–80 s showed a significant increase (up to 500%) at 5 min and beyond.

#### 2.4.3. Optimization of Sample Amount

Under fixed conditions (incubation temperature: 45 °C, incubation time: 5 min, injection volume: 500 μL), different sample amounts (0.15, 0.20, 0.25, 0.30, and 0.35 g) were evaluated in triplicate. The results demonstrated that 0.25 g provided the most satisfactory chromatographic profiles, and thus, this amount was selected for further analysis.

#### 2.4.4. Optimization of Injection Volume

Precisely weighed 0.25 g of sample R5 was analyzed at 45 °C for 5 min while varying the injection volume (200, 300, 400, 500, and 600 μL). Each condition was tested in triplicate. The chromatographic peaks stabilized when the injection volume reached 500 μL, indicating odor saturation. Therefore, 500 μL was chosen as the optimal injection volume.

### 2.5. Determination of Detection Conditions

Through single-factor experiments, the Heracles NEO detection conditions for raw and bran-fried samples were determined as follows: a sample vial volume of 20 mL, sample weight of 0.25 g, incubation temperature of 45 °C, incubation time of 5 min, incubation oven rotation speed of 250 r/min, trap initial temperature of 40 °C, trap final temperature of 250 °C, trap split flow rate of 10 mL/min, trap duration of 14 s, injection port temperature of 200 °C, injection port pressure of 10 kPa, injection volume of 500 μL, injection rate of 125 μL/s, injection time of 9 s, valve temperature of 250 °C, initial column temperature of 50 °C, column temperature program of 1.0 °C/s to 80 °C, then 3.0 °C/s to 250 °C (held for 21 s), acquisition time of 110 s, FID gain of 12. The optimal detection conditions for RATR and BATR samples are systematically summarized in Table 2.

### 2.6. Analysis of Ultra-Fast Gas-Phase Electronic Nose Detection

Following the detection conditions specified in Section 2.4, the raw and bran-fried ATR slice samples from each batch were analyzed to establish odor fingerprint spectra. The calibration mixture of n-alkanes (nC6~nC16) and reference standards was placed in headspace vials and analyzed under the same incubation and analysis conditions as the samples. The retention times of the chromatographic peaks of raw and bran-fried ATR slices were converted into Kovats retention indices. Qualitative analysis of the volatile components in the samples was conducted by referencing the AroChemBase database [22], and changes in odor components between raw and bran-fried ATR slices were compared. Multivariate statistical analysis of the odor fingerprint spectra of each sample was performed using Alpha Soft 17.0 software (Toulouse, France) to compare sample differences and identify distinctive odor marker compounds. Calibration was performed using the n-alkane standard solution (nC6 to nC16), converting the retention times into retention indices, and then qualitative identification was carried out using the Kovats retention index. Compounds were qualitatively analyzed in the Arochembase database [23].

### 2.7. E-Eye Analysis

After turning on the instrument (C17600 Portable Spectrophotometer, X-Rite Color Technology Co., Ltd., Grand Rapids, MI, USA; Software Version: ColorMaster™ SW v5.2.1), the 24-color calibration plate was placed in the instrument for calibration. The lens exposure and focal length were adjusted to appropriate settings, using a 5 nm aperture, D65 light source, and both top and bottom illumination. Images were captured in single snapshot mode. The raw and bran-fried ATR samples, which had been ground and sieved through a No. 5 sieve, were evenly placed in a Petri dish for image acquisition. The average value was taken after three parallel measurements, and the color codes and proportions of each sample were recorded. Each color code was represented by three indicators: lightness value (L^*^), red-green value (a^*^), and yellow-blue value (b^*^) [24]. The L* value ranges from 0 to 100, corresponding to colors from black to white; a^*^ ranges from positive to negative, corresponding to colors from red to green; and b^*^ ranges from positive to negative, corresponding to colors from yellow to blue. The total color value (E^*^_ab_) was calculated using the formula E^*^_ab_ = [(L^*^)^2^ + (a^*^)^2^ + (b^*^)^2^]^1/2^. The greater the value of E^*^_ab_, the lighter the color appeared. In addition, the total color difference (ΔE^*^_ab_) could be used to represent the color difference between two color codes. although it could not indicate the color bias. The calculation formula is as follows: E^*^_ab_ = [(L^*^)^2^ + (a^*^)^2^ + (b^*^)^2^]^1/2^ [25].

### 2.8. E-Tongue Analysis

The taste characteristics of ATR samples were analyzed with an S-5000Z taste sensor, E-tongue instrument (INSENT Inc., Atsugi-shi, Japan) that was equipped with five chemical sensors for bitterness and aftertaste bitterness (aftertaste-B), astringency and aftertaste astringency (aftertaste-A), umami and richness, saltiness, and sourness, as well as two reference electrodes [26]. The ATR slices were pulverized and passed through a No. 3 sieve. 2 g of the powdered sample was weighed, mixed with 100 mL of purified water, thoroughly stirred, and sonicated for 10 min. Samples were maintained at 25 ± 0.5 °C during extraction. The mixture was then centrifuged at 3000 r/min for 5 min, and the supernatant was filtered through filter paper to obtain the test solution [27].

The reference solution (KCl/tartaric acid mixture) served as the zero baseline, with tasteless points set at 0 for all taste indicators except sourness (–13) and saltiness (–6), which exhibited inherent background signals. Only taste values exceeding these thresholds were considered analytically significant. The taste detection threshold was defined by the reference solution (KCl/tartaric acid mixture), establishing baseline values of –13 (sourness) and –6 (saltiness). Values exceeding these thresholds were considered detectable tastes. All experiments were performed in triplicate.

### 2.9. Data Analysis

The collection of electronic nose data was performed using the Heracles NEO ultra-fast gas chromatograph (Alpha MOS, Toulouse, France) with the following analytical protocol: (1) Chromatographic separation was conducted on dual MXT-5 columns (10 m × 0.18 mm × 0.4 μm) with hydrogen carrier gas, and calibration was established using n-alkane standards (C6-C16, Restek); (2) compound identification was achieved through the integrated AroChemBase 2023 (Heracles edition) with stringent matching criteria, further validated by NIST 2020 mass spectral library; and (3) multivariate statistical analysis (AlphaSoft 17.0) included PCA with Pareto scaling and DFA with 7-fold cross-validation. All experiments were conducted in triplicate, with results expressed as mean ± standard deviation (SD). In this study, chemometric analysis was conducted using the Alphasoft 17.0 software integrated with the Heracles Neo ultra-fast gas-phase electronic nose.

## 3. Results

### 3.1. Establishment of Odor Fingerprint Spectra

**Figure 1:**
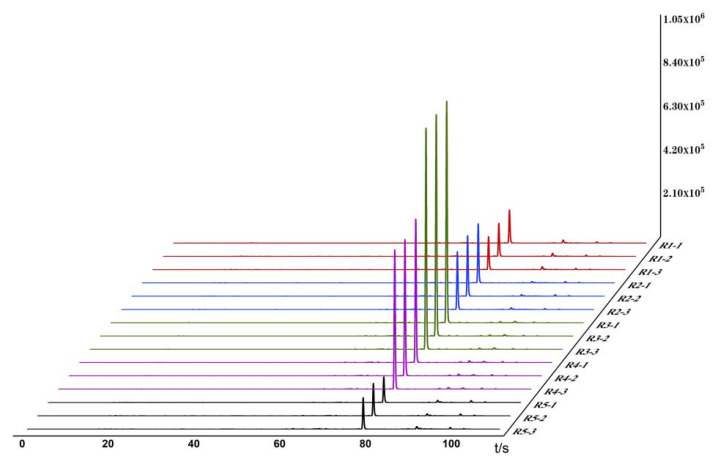
Odor fingerprints of 5 batches of RATR.

**Figure 2:**
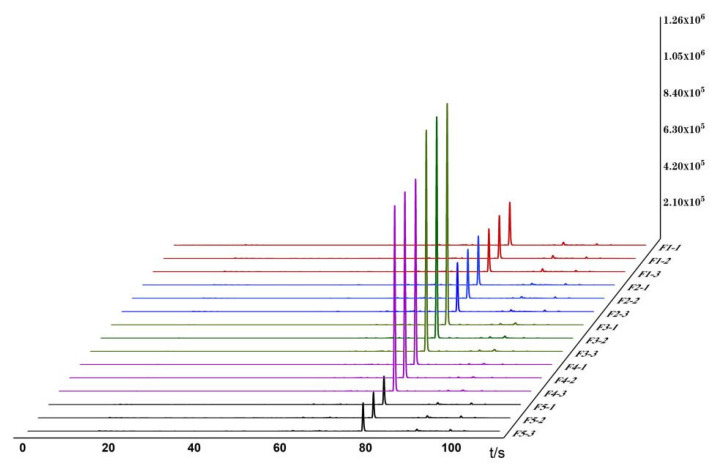
Odor fingerprints of 5 batches of BATR.

Based on the detection and analysis results from the Heracles NEO ultra-fast gas-phase electronic nose, odor fingerprint spectra for raw and bran-fried ATR slice samples were established using signals collected from the MXT-5 column. The results are shown in Table 3 and Figure 1 and Figure 2. *** *p* < 0.001 (extremely significant), ** *p* < 0.01 (very significant), * *p* < 0.05 (significant).

In the images, different colors represent different samples: red represents Hunan; blue represents Sichuan; green represents Jiangxi; purple represents Guizhou; and black represents Hubei. From the overlapping gas chromatograms, it can be observed that the detection results of the two groups of samples are generally similar, with the peaks of higher response values having nearly identical retention times but significant differences in peak areas. From the spectra, the chromatographic peaks are generally higher between 70 and 80 s, with a characteristic peak near 80 s. Analysis of this peak reveals that the chromatographic peak of the samples from Jiangxi is significantly higher than those from the other four regions. Between 80 and 100 s, the chromatographic peaks of all groups are relatively low, with no significant differences among the groups. Analysis of the original spectra shows that the differences between raw and bran-fried ATR are mainly reflected in changes in peak height. In terms of peak height differences, the chromatographic peak heights of bran-fried ATR from all five regions are significantly increased compared to the raw samples, with the most noticeable difference observed in the samples from Hunan before and after processing.

### 3.2. Analysis of Odor Changes

The Heracles NEO ultra-fast gas-phase electronic nose can provide sensory information on volatile components. By comparing the gas chromatograms of ATR samples before and after processing on the MXT-5 column, as shown in Figure 1 and Figure 2, and combining the information from the Arochembase database, qualitative analysis results were presented in Table 4.

The results showed that raw ATR exhibited 12 odor components, while bran-fried ATR exhibited 15 odor components. Among these, 12 odor components were common to both, including benzaldehyde, o-dichlorobenzene, L-limonene, undecane, 2,6-dimethoxyphenol, decanal, geranial, methyl eugenol, 2-dodecanol, methyl dodecanoate, 4-undecalactone, and tributyl phosphate, which have odors such as almond and bitterness. Additionally, bran-fried ATR exhibited three new odor components—methanol (alcoholic/pungent notes), 2-propanol (acetone/alcohol-like characteristics), and 2-cyclopentenone (/)—among which the first two components collectively contributed to sharp, penetrating aroma consistent with the traditional description of the roasted fragrance produced after bran-frying [28]. Formal sensory evaluation and GC-MS analysis of aroma characteristics will be conducted in subsequent studies.

### 3.3. Principal Component Analysis (PCA) of the Heracles NEO Electronic Nose

**Figure 3:**
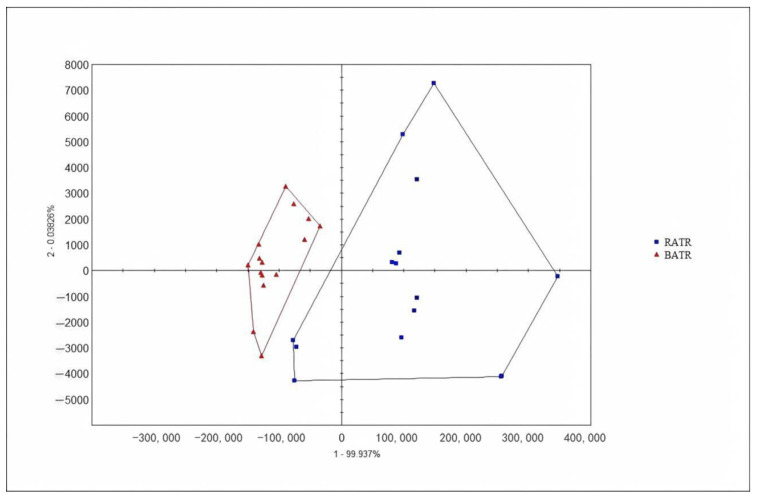
PCA of raw and bran-fried ATR.

**Figure 4:**
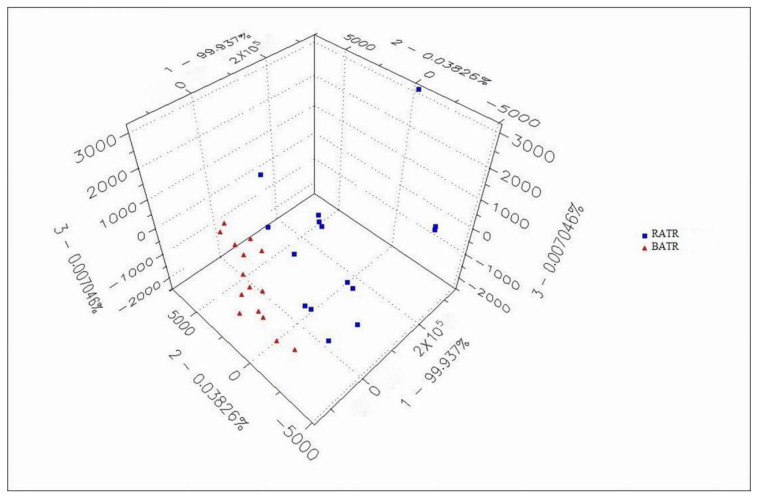
3D PCA of raw and bran-fried ATR.

The chromatographic peak areas, normalized to total ion chromatogram (TIC) area, were used as variables for principal component analysis (PCA), and the results are shown in Figure 3 and Figure 4. In the PCA model, the contribution rate of the first principal component (PC1) was 99.937%, the second principal component (PC2) was 0.03826%, and the third principal component (PC3) was 0.007046% [29]. The cumulative contribution rate of the principal components reached 99.9823%, effectively distinguishing between raw and bran-fried ATR.

PCA of raw and bran-fried ATR samples demonstrated that the Heracles NEO ultra-fast gas-phase electronic nose can rapidly distinguish between raw and bran-fried processed samples of ATR.

### 3.4. Discriminant Factor Analysis (DFA) of the Heracles NEO Electronic Nose

**Figure 5:**
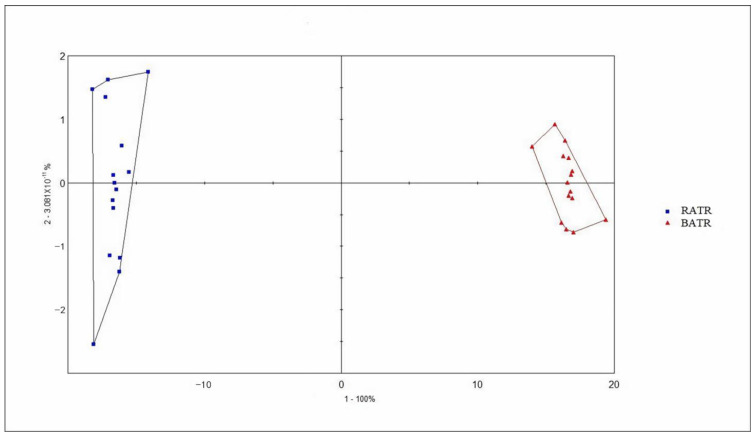
DFA of raw and bran-fried ATR.

Discriminant Function Analysis (DFA) is a statistical method that optimizes class separation by maximizing inter-group differences while minimizing intra-group variations based on training datasets with two or more predefined categories, thereby establishing a well-discriminated model to characterize sample differentiation [30]. Building upon Principal Component Analysis (PCA), DFA enhances the discrimination capability by compressing within-group variance and amplifying between-group differences, which facilitates clearer differentiation between raw and bran-fried *Acori tatarinowii Rhizoma* (ATR) samples. As demonstrated in Figure 5, the complete separation and significant distance between raw and processed ATR clusters confirm the superior discriminative power of DFA in characterizing their distinct odor profiles.

### 3.5. E-Eye Analysis

**Figure 6:**
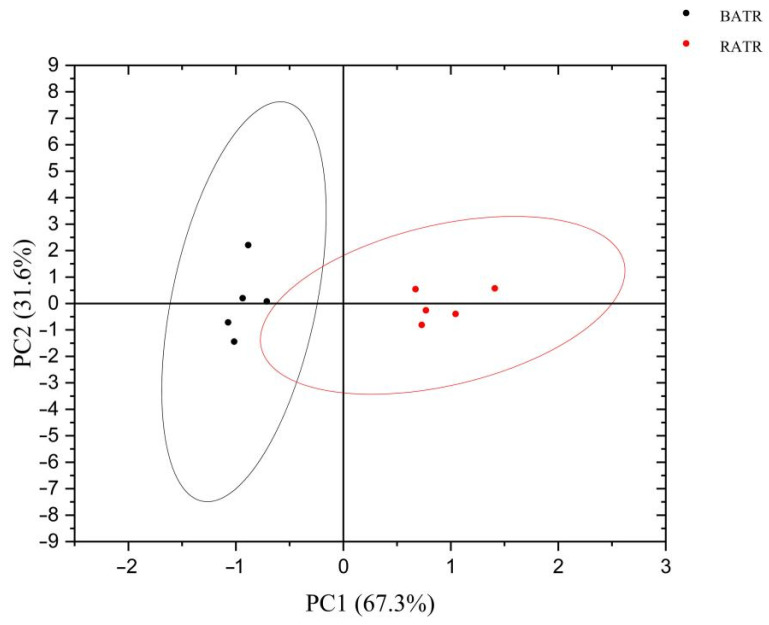
PCA of E-eye between raw and bran fried ATR.

The lightness values of RATR range from 58.65 to 64.85, indicating that the raw samples are relatively light in color. The lightness values of BATR range from 48.91 to 53.50, showing a significant decrease compared to the raw samples. This suggests that bran frying darkens the color of the samples. The a^*^ values range from 5.83 to 6.41, indicating a slight red dominance. The a^*^ values range from 7.41 to 8.34, showing an increase in red dominance compared to the raw samples. This suggests that bran-frying enhances the red component of the color. The b^*^ values range from 13.95 to 15.22, indicating a slight yellow dominance. The b^*^ values range from 13.79 to 17.29, showing a slight increase in yellow dominance compared to the raw samples. The E^*^_ab_ values range from 78.48 to 85.74. The E^*^_ab_ values range from 70.11 to 79.13. The overall color difference is lower for bran-fried samples, indicating a more consistent color profile after processing. This suggests that bran-frying also enhances the yellow component of the color. The chromaticity values of samples R1 to R5 and B1 to B5 are presented in Table 5, and PCA of the electronic eye are shown in Figure 6. Significance analysis: one-way ANOVA with Tukey’s post hoc test. The bran frying process significantly (*p* < 0.05) reduced lightness (L^*^) and increased redness (a^*^), while yellowness (b^*^) remained unchanged. The total color difference (E^*^_ab_) decreased by 9.1%, confirming visible color modification. ** *p* < 0.01, *** *p* < 0.001. All data are presented as mean ± standard deviation (*n* = 3).

### 3.6. Correlation Analysis of E-Nose and E-Eye

**Figure 7:**
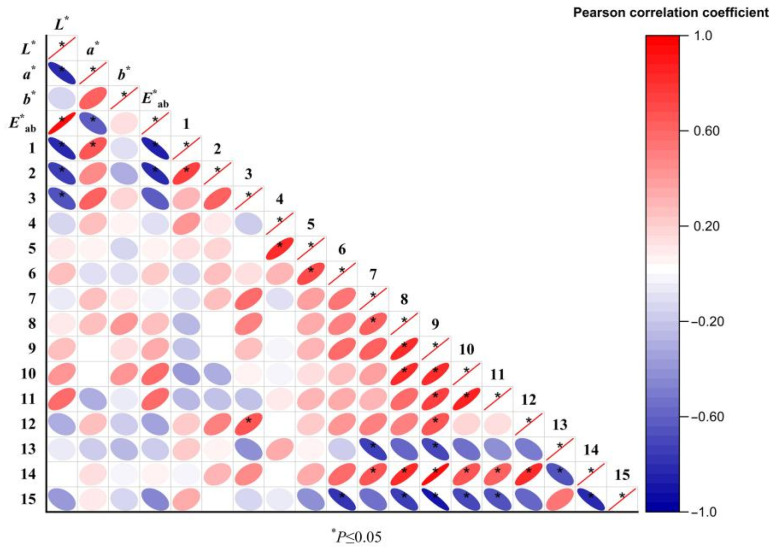
Heat map of Pearson correlation coefficient between chromaticity values and odor components of raw and bran-fried ATR.

A correlation analysis was conducted between the chromaticity and chemical components of odor in ATR before and after stir-frying with bran. The peak areas of 15 odor components and the corresponding chromaticity parameters (L^*^, a^*^, b^*^, E^*^_ab_) were input into Origin Pro 2021 software to generate a visualized heatmap. The results are presented in Figure 7. It was observed that odor components 1, 2, and 3 exhibited significant correlations with chromaticity values, all showing negative correlations with L. These findings are consistent with the results obtained from the electronic nose analysis.

### 3.7. E-Tongue Analysis

**Figure 8:**
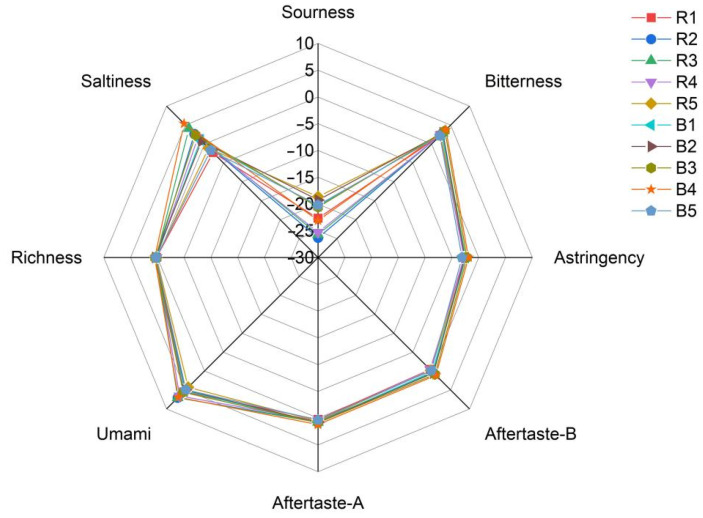
Radar fingerprint of E-tongue data between RATR and BATR.

The acidity of the raw samples is consistently negative, with relatively high absolute values ranging from −18.65 to −26.31, indicating a low level of acidity. The bitterness values are all positive, ranging from 2.23 to 2.95, suggesting a moderate bitter taste in these samples. Astringency values are also negative, ranging from −2.39 to −2.75, indicating a weak astringent sensation. The aftertaste values are close to zero, implying a subtle aftertaste. Umami values are positive, ranging from 4.28 to 7.06, indicating a noticeable umami taste. The richness values are low, ranging from 0.11 to 0.22, suggesting a light and delicate mouthfeel. Saltiness values vary between positive and negative, but with small absolute values, indicating an indistinct salty taste. After bran-frying, the acidity remains negative, with values ranging from −19.27 to −23.09, but the absolute values are slightly lower than those of the raw samples, indicating a marginally higher acidity compared to the raw state. Bitterness values are all positive, ranging from 2.14 to 3.73, and generally higher than those of the raw samples, suggesting an enhanced bitter taste post-processing. Astringency values remain negative, ranging from −1.91 to −3.14, but with smaller absolute values, indicating a slightly stronger astringent sensation. Aftertaste values are higher, particularly for Aftertaste-A, indicating a more pronounced aftertaste. Umami values remain positive, ranging from 4.89 to 6.89, similar to those of the raw samples. Richness values are higher, ranging from 0.23 to 0.49, suggesting a more complex mouthfeel. Saltiness values vary between positive and negative, but with larger absolute values, indicating a more noticeable salty taste in some samples. In summary, the raw samples exhibit low acidity, moderate bitterness, weak astringency, a subtle aftertaste, and a light mouthfeel. In contrast, the bran-fried samples show slightly higher acidity, stronger bitterness, slightly stronger astringency, a more pronounced aftertaste, and a richer mouthfeel. The results are presented in Table 6 and Figure 8. Statistical methods: two-tailed t-test; significance levels: *p* < 0.05 indicates a statistically significant difference; *p* < 0.01 indicates a highly statistically significant difference. Bran-fried significantly enhanced bitterness (*p* = 0.008), aftertaste characteristics (*p* < 0.05), and richness (*p* = 0.001) while reducing sourness (*p* = 0.042). The umami and astringency profiles remained stable, suggesting selective modification of taste attributes during processing.

## 4. Discussion

Currently, there is extensive research on the chemical components of ATR, but studies on its odor components, color, and taste before and after bran stir-frying are relatively limited. This study developed a differential analysis method for the odor of raw and bran-stir-fried ATR slices using the Heracles NEO ultra-fast gas-phase electronic nose [31]. The volatile components in five batches of raw and bran-fried ATR samples were analyzed. The odor fingerprint spectra of raw and bran-stir-fried ATR slices were determined, and 15 odor chemical components were identified. Among these, methanol, 2-propanol, and 2-cyclopentenone were newly identified odor components after bran stir-frying. Combined with chromaticity measurements using an electronic eye, a correlation analysis between odor components and chromaticity values was conducted, further confirming methanol, 2-propanol, and 2-cyclopentenone as differential odor components between raw and bran-stir-fried ATR. Volatile components are the main active constituents responsible for the dampness-resolving effect of ATR. Previous studies have found that the main volatile components, such as β-asarone, α-asarone, and trans-methylisoeugenol, increase in content after bran stir-frying [32]. It is hypothesized that the enhanced dampness-resolving and stomach-opening effects of bran-stir-fried ATR may be related to the increase in the types and content of these volatile components [33]. However, the underlying mechanisms require further investigation through modern pharmacological studies.

Based on the E-eye analysis, bran frying results in a noticeable darkening of the samples, an increase in red dominance, and a slight increase in yellow dominance [34]. The bran-fried samples exhibit a more consistent color profile, as indicated by the lower range of E*_ab_ values compared to the raw samples. This analysis provides valuable insights into the color changes associated with the bran-frying process, which can be crucial for quality control and standardization in the production of ATR slices.

The E-tongue analysis results show that raw samples exhibit low acidity, moderate bitterness, weak astringency, a subtle aftertaste, and a light mouthfeel. In contrast, the bran-fried samples show slightly higher acidity, stronger bitterness, slightly stronger astringency, a more pronounced aftertaste, and a richer mouthfeel. This provides technical support for identification and quality control of RATR and BATR [35].

Based on the integrated analysis of Heracles NEO, E-eye, and E-tongue data, this study establishes the following measurable quality standards for bran-fried ATR (BATR) authentication and quality control: (1) Volatile Markers: mandatory detection of methanol, 2-propanol, and 2-cyclopentenone; (2) Color Specifications CIELAB ranges: L^*^ 48.91–53.50, a^*^ 7.41–8.34, minimum color difference: ΔE^*^_ab_ ≥ 15.0 vs. raw ATR; (3) Taste Modulation: bran frying induced a 10% reduction in sourness intensity (raw: −26.31 to −18.65; processed: −23.09 to −19.27) and enhanced saltiness in select batches (30% increase), demonstrating significant taste change. Changes in the composition and content of volatile components during the stir-frying process are the material basis for the altered properties and efficacy of bran stir-fried ATR. Further pharmacological validation is required to establish the bioactivity implications. Future studies should systematically investigate (1) the kinetic patterns of component changes during bran stir-frying using real-time mass spectrometry monitoring and (2) the mechanism–processing–efficacy relationships through integrated pharmacodynamic and metabolomic approaches. This research provides a feasible strategy for identifying ATR before and after processing, addressing the limitations of traditional morphological identification. It also offers reference value for the application of sensory technologies such as E-nose, E-eye, and E-tongue in the detection of other food and pharmaceutical products. By analyzing the odor, color, and taste of RATR and BATR, this study lays a solid foundation for research on the quality standards and optimization of processing techniques for ATR slices. Additionally, it provides a reference for further studies combining pharmacological research to clarify the pharmacodynamic material basis and elucidate the processing mechanisms of bran-stir-fried ATR. However, we acknowledge the limitation of our research and will incorporate key model robustness metrics—including classification accuracy, cross-validation results, and receiver operating characteristic analysis—in future validation studies with expanded sample sizes to further substantiate the predictive performance of our model.

## 5. Conclusions

This study demonstrates that bran stir-frying significantly alters the odor, color, and taste profiles of ATR slices, with newly identified volatile components (methanol, 2-propanol, and 2-cyclopentenone) contributing to the differential odor. The darkening, increased redness, and enhanced bitterness/astringency of bran-fried ATR correlate with its traditional efficacy in dampness-resolving and stomach-opening effects. The integration of E-nose, E-eye, and E-tongue technologies provides a robust method for distinguishing raw and processed ATR, offering valuable insights for quality control and standardization. Further pharmacological research is needed to elucidate the mechanisms underlying these changes and optimize processing techniques. This approach also holds promise for applications in other food and pharmaceutical products.

## Figures and Tables

**Table 1:** Sample source information of ATR.

Serial	Origin	Serial	Origin	Batch
R1	Hunan	B1	Hunan	20231024004
R2	Sichuan	B2	Sichuan	20231024013
R3	Jiangxi	B3	Jiangxi	20231024001
R4	Guizhou	B4	Guizhou	20231024010
R5	Hubei	B5	Hubei	20231024007

**Table 2:** Heracles NEO ultra-fast gas-phase electronic nose experimental parameters.

Condition	Parameter	Condition	Parameter
Sample vial	20 mL	Trap temperature	40 °C
Sample amount	0.25 g	Column front pressure	57 kPa
Injection volume	500 μL	Trap time	14 s
Incubation temperature	45 °C	Valve temperature	250 °C
Incubation time	5 min	Initial column oven temperature	50 °C
Injection speed	125 μL/s	Column temperature program	3.0 °C/s
Injection time	9 s	Acquisition time	110 s
Inlet temperature	200 °C	FID gain	12
Inlet pressure	10 kPa	FID offset	1000

**Table 3:** Differences in a relative peak area before and after bran-frying.

No.	Relative Peak Area	No.	Relative Peak Area	Origin
R1	(1.47 ± 0.05) × 10^5^	B1	(3.86 ± 0.12) × 10^5^ ***	Hunan
R2	(2.87 ± 0.08) × 10^5^	B2	(3.22 ± 0.09) × 10^5^ *	Sichuan
R3	(7.59 ± 0.15) × 10^5^	B3	(12.78 ± 0.25) × 10^5^ ***	Jiangxi
R4	(6.94 ± 0.12) × 10^5^	B4	(7.49 ± 0.13) × 10^5^	Guizhou
R5	(1.24 ± 0.03) × 10^5^	B5	(1.64 ± 0.04) × 10^5^ *	Hubei

**Table 4:** Possible compounds and sensory description information in raw and bran-fried ATR.

No.	Molecular Formula	t_R_/s	RI	Possible Compound	Similarity Index	Odor Information	RATR	BATR
1	CH_4_O	16.63	436	Methanol	91.06	Alcoholic; spicy, etc.	−	+
2	C_3_H_8_O	19.25	489	2-Propanol	89.63	Acetone; alcohol; ethanol, etc.	−	+
3	C_5_H_6_O	50.28	842	2-Cyclopentenone	74.89	/	−	+
4	C_7_H_6_O	61.84	968	Benzaldehyde	91.21	Almond; bitter, etc.	+	+
5	C_6_H_4_Cl_2_	67.68	1041	o-Dichlorobenzene	88.15	Aromatic; aromatic hydrocarbon odor	+	+
6	C_10_H_16_	68.04	1051	L-Limonene	91.13	Orange; mint, etc.	+	+
7	C_11_H_24_	71.72	1106	Undecane	81.56	Alkane; fusel alcohol, etc.	+	+
8	C_8_H_10_O_3_	77.15	1197	2,6-Dimethoxyphenol	87.03	Sesame oil; phenol, etc.	+	+
9	C_10_H_20_O	78.30	1219	Decanal	98.35	Aldehyde; candle, etc.	+	+
10	C_10_H_16_O	81.23	1274	Geranial	96.69	Orange; mint, etc.	+	+
11	C_9_H_12_O_3_	83.02	1310	Methyl eugenol	85.42	Aromatic; spicy odor	+	+
12	C_12_H_26_O	88.67	1427	2-Dodecanol	93.53	Coconut; candle, etc.	+	+
13	C_13_H_26_O_2_	92.97	1521	Methyl dodecanoate	95.87	Coconut; cream, etc.	+	+
14	C_11_H_20_O_2_	94.93	1562	4-Undecanolide	92.79	Apricot; coconut; peach, etc.	+	+
15	C_12_H_27_O_4_P	98.61	1639	Tributyl phosphate	80.19	Odorless	+	+

“RI” represents retention index; “+” indicates the detected component; “−” indicates that the component is not detected.

**Table 5:** Chromatic values of raw and bran fried ATR.

No.	Chromatic Values			
	L^*^	a^*^	b^*^	E^*^_ab_
R1	61.01 ± 0.12	5.83 ± 0.05	14.17 ± 0.08	81.01 ± 0.15
R2	59.97 ± 0.09	6.41 ± 0.07 *	15.22 ± 0.11 *	81.60 ± 0.13
R3	64.85 ± 0.15 **	6.20 ± 0.04 *	14.69 ± 0.09	85.74 ± 0.17 **
R4	60.11 ± 0.11	6.33 ± 0.06 *	14.24 ± 0.07	80.68 ± 0.12
R5	58.65 ± 0.08	5.88 ± 0.03	13.95 ± 0.05	78.48 ± 0.10
B1	52.19 ± 0.18 **	7.59 ± 0.12 ***	15.16 ± 0.15	74.94 ± 0.22 ***
B2	49.69 ± 0.15 ***	7.82 ± 0.10 ***	14.41 ± 0.13 *	71.92 ± 0.19 ***
B3	53.50 ± 0.20 ***	8.34 ± 0.14 ***	17.29 ± 0.18 ***	79.13 ± 0.25 **
B4	48.91 ± 0.14 ***	7.41 ± 0.09 ***	13.79 ± 0.12	70.11 ± 0.17 ***
B5	51.23 ± 0.16 ***	7.88 ± 0.11 ***	15.31 ± 0.14	74.42 ± 0.20 ***

All values represent mean ± SD (*n* = 3), * *p* < 0.05, ** *p* < 0.01, *** *p* < 0.001.

**Table 6:** E-tongue data of raw and bran fried ATR.

NO.	Type	Sourness	Bitterness	Astringency	Aftertaste-B	Aftertaste-A	Umami	Richness	Saltiness
0	Tastless	−13	0	0	0	0	0	0	−6
1	R1	−22.68 ± 0.85 *	2.23 ± 0.12	−2.65 ± 0.08	−0.44 ± 0.05	0.24 ± 0.03	5.53 ± 0.25 **	0.17 ± 0.01	−2.31 ± 0.15
2	R2	−26.31 ± 1.10 **	2.95 ± 0.15 *	−2.58 ± 0.07	0.46 ± 0.04 *	0.63 ± 0.05 **	7.05 ± 0.35 ***	0.17 ± 0.01	2.64 ± 0.20
3	R3	−25.72 ± 1.05 **	2.89 ± 0.14 *	−2.41 ± 0.06 *	0.33 ± 0.03 *	0.78 ± 0.06 ***	7.06 ± 0.30 ***	0.22 ± 0.02 *	4.07 ± 0.25 **
4	R4	−25.27 ± 0.95 **	2.33 ± 0.10	−2.75 ± 0.09	−0.39 ± 0.04	0.51 ± 0.04 **	6.55 ± 0.28 ***	0.11 ± 0.01	1.72 ± 0.12
5	R5	−18.65 ± 0.75 *	2.35 ± 0.11	−2.39 ± 0.05 *	−0.20 ± 0.02	0.56 ± 0.04 **	4.28 ± 0.20 **	0.14 ± 0.01	−1.11 ± 0.08
6	B1	−20.42 ± 0.92 *	2.65 ± 0.13 *	−2.47 ± 0.12	0.19 ± 0.02 *	0.80 ± 0.06 ***	5.25 ± 0.26 **	0.25 ± 0.02 **	1.02 ± 0.08 *
7	B2	−19.27 ± 0.87 *	2.94 ± 0.15 *	−2.61 ± 0.13	0.49 ± 0.04 **	0.82 ± 0.06 ***	4.99 ± 0.25 **	0.36 ± 0.03 ***	0.76 ± 0.06 *
8	B3	−20.60 ± 0.93 *	3.47 ± 0.17 **	−2.19 ± 0.11 *	0.87 ± 0.07 ***	0.85 ± 0.06 ***	5.61 ± 0.28 **	0.40 ± 0.03 ***	2.33 ± 0.17 **
9	B4	−23.09 ± 1.04 **	3.73 ± 0.19 **	−1.91 ± 0.10 **	1.11 ± 0.09 ***	1.21 ± 0.09 ***	6.89 ± 0.34 ***	0.49 ± 0.04 ***	5.35 ± 0.38 ***
10	B5	−20.21 ± 0.91 *	2.14 ± 0.11	−3.14 ± 0.16	−0.12 ± 0.01	0.34 ± 0.03 *	4.89 ± 0.24 **	0.23 ± 0.02 **	−1.69 ± 0.12

All data were recorded as absolute output values referenced against artificial saliva (reference solution), with the electronic tongue’s artificial saliva state simulating the oral cavity condition containing only saliva. All values represent mean ± SD (*n* = 3), * *p* < 0.05, ** *p* < 0.01, *** *p* < 0.001.

## Data Availability

The original contributions presented in this study are included in the article. Further inquiries can be directed to the corresponding authors.

## Abbreviations

The following abbreviations are used in this manuscript:

ATR	*Acori tatarinowii Rhizoma*
VOCs	Volatile organic compounds
BATR	Bran-fried *Acori tatarinowii Rhizoma*
RATR	Raw *Acori tatarinowii Rhizoma*
